# Supplementary material for: What do editors of medical journals think about opportunities and barriers to advancement in the publication of plain language summaries? A qualitative analysis
Source: PLoS One. 2026 Mar 11;21(3):e0343058. doi: 10.1371/journal.pone.0343058 (PMC12978476; doi:10.1371/journal.pone.0343058)
Supplement: S1 Text — (DOCX) [file pone.0343058.s001.docx]

**Interview schedule**

*Note: This document provides example interview topics and questions that will be addressed. In keeping with qualitative methods, we expect that this topic guide will continually evolve before and during the study in response to emerging learnings. Additional probing questions may be asked to clarify participant responses and ensure understanding.*

The interviews will adhere to the following pattern:

1. Introduction
2. Overview of the topic and study rationale
3. Interview questions

Here is an example of a typical introduction:

Good morning/afternoon. Thank you for taking the time to talk to me about your experience at (insert journal) with plain language summaries, which we define as being short summaries of research articles written for a general audience, using plain, easy-to-understanding language.

I’m Karen Gainey and this study is the final project in my PhD with the Sydney Health Literacy Lab at the University of Sydney. My research involves the use of plain language summaries as a way of communicating health information to a general audience. My previous research has focused on guidelines for writing plain language summaries and understanding what end users of plain language summaries want from them.

You were invited to participate in this study because of your editorial role with (insert journal). I’ll be asking you questions to help me understand more about how the editorial process works, particular in relation to plain language summaries and any barriers that might exist to how they are published. Keep in mind that we're just as interested in negative comments about lay summaries and health information as positive comments, and at times the negative comments are the most helpful.

You've probably noticed the ‘recording’ icon on your screen, so a reminder that we're recording the audio from the session via ZOOM because we don't want to miss any of your comments. We won’t be recording any video nor will we be saving the chat transcript. You may be assured of complete confidentiality.

**Interview questions**

I’d like to understand the factors that impact how decisions are made by health and medical journals that relate to plain language summaries (PLSs). I appreciate that some decisions may be made by the publishing group, rather than each journal, so it would be helpful for me to better understand how the process works.

***Decision making and how that could impact changes to PLSs***

- Can you tell me about your role as a journal editor?
- Can you tell me about an experience you have had with PLSs?
- When it comes PLSs, who makes decisions about them?
  - Length
  - Content
  - Structure
  - Format i.e., text-based only or graphical/video options
- Have you faced any barriers to the implementation of PLSs?

***Mandatory vs optional PLSs***

I know from my previous study that PLSs are not mandatory for all journals, so I’d like to explore factors that might have led your journal’s decision to make PLSs mandatory or optional.

- Are PLSs mandatory for your journal?
  - What is the reason behind your decision? i.e., what factors were considered?
  - Does your journal encourage authors to include a PLS even if they are not mandatory? If so:
    - How do you encourage authors to write PLSs?
    - Do you think it has increased the number of PLSs submitted?

***PLS guidelines***

- - Does your journal have any guidelines to support authors in writing PLSs?
  - Who developed them? Can you tell me a bit about that process?
  - Was there any end-user input in their development?
  - When were they last reviewed?

***PLS guidelines and compliance***

My previous project was a study comparing journal guidelines for writing PLSs against PLSs for a number of health and medical journals. We wanted to know if PLSs were written in accordance or in compliance with, the PLS guidelines for the journal in which they were published.

- - Do you know if anyone has even done this kind of check with the PLSs for your journal to see if they are written according to your PLS guidelines?
  - If checked, at what stage of submission process and by who? What are the practical barriers to this?
  - What action, if any, was taken for any non-compliance?
  - If checked, what items were the most non-compliant?
  - How important would you rate checking for compliance and why?

***PLS audience***

- - What audience(s) do you design your PLSs for and why? i.e., what factors went into the decision.
  - Is that reflected in your PLS guidelines? i.e., in the PLS guidelines, is it clear who the audience is?

***Access to PLSs***

- - How are PLSs in your journal made accessible to readers?
  - What are your thoughts on access to PLSs? e.g., for those articles behind paywalls. What are the barriers and facilitators to access?
  - What are your thoughts on providing a separate section on your journal website just for PLSs, searchable with keywords? e.g., like the Cochrane Database

***PLS and peer review***

- - Are PLSs included formally in the peer review process? i.e., part of a checklist or reporting requirement?
  - If not, why not? (could be that they are not mandatory for that journal)
  - If they are included, are there guidelines for peer reviewing PLSs?
  - Do peer reviewers tend to include PLSs in the report even if not required?
  - How important would you rate peer reviewing PLSs and why?

***Outsourced PLSs***

- - Does your journal offer an outsourcing service for PLSs?
  - How popular is this with authors?
  - Do you have any feedback from authors on this service?

***PLS formats***

- - What formats does your journal offer? i.e., text-based, graphical, video.
  - If not text-based are available, how popular are they?
  - Have you had any feedback on the use of non text-based formats?

***Use of artificial intelligence (AI) tools***

- - What are your thoughts on the use of AI tools for writing PLSs?
  - Does your journal have guidelines for the use of AI in general or PLSs specifically?

***Future of PLSs***

- - What do you think is the future of PLSs in health and medical publishing?
  - What factors will impact changes to how PLSs are written, published or made available?

***Wrapping up***

- Are there any other barriers or facilitator related to the publication or dissemination of PLSs that you would like to reflect on and share today?
- Of all the aspects of PLSs we’ve discussed today, which do you think are the most and least important, and why?
- Is there anything else of note we haven’t covered that you would like to comment on?
