# Supplementary material for: What do editors of medical journals think about opportunities and barriers to advancement in the publication of plain language summaries? A qualitative analysis
Source: PLoS One. 2026 Mar 11;21(3):e0343058. doi: 10.1371/journal.pone.0343058 (PMC12978476; doi:10.1371/journal.pone.0343058)
Supplement: Attachment — Submitted filename: PONE-D-25-30641 peer review report.docx [file pone.0343058.s002.docx]

PONE-D-25-30641

This qualitative analysis provides insight into the thoughts and impressions of peer-reviewed journal editors on the adoption of plain-language summaries (PLSs) by medical journals, which is a topic of ongoing debate in terms of how to encourage greater adoption of this innovative format by both journals and authors.

Overall, I think that the manuscript is deserving of publication and offers an opportunity to further contribute to the discussion about the implementation of PLSs. However, there are a few comments that I hope that Authors this manuscript may be able to consider and apply before I believe this manuscript would be fit for publication. For ease, I have simply commented by section rather than splitting my comments in major/minor categories below:

Introduction

1. Would it be possible to mention the heterogeneity of what may be considered a PLS? The term itself has become anything but plain language, given that the likes of the Good Publication Practice Guidelines 2022 offer a very broad definition of just about any derivative of a manuscript, while the lay person would probably consider a PLS to be a version of the abstract written in plain language. I think this is particularly relevant in that the impression I get is that this manuscript intends to focus on written PLSs, excluding video podcasts etc.
2. Page 10, line 16 – Is the Stoll reference intended to be replaced with a reference number?

Methods

1. Were there any additional limitations on eligibility for this study? In particular, I think it might be pertinent to mention if the subjects could only represent journals publishing in English, given the multinational representation, and this seems to be mentioned in the Discussion on page 40, lines 828–829.
2. Table 1, Sample specificity row – Is sample density correct here, when referring to sample specificity? As a casual reader, I would interpret high sample density as suggesting a high proportion of journals who were eligible for this study were sampled.
3. Table 1, Established theory row – I’m not sure I 100% understand how the application in the current study links to the need for a smaller sample size. I guess I was kind of looking for an explanation of how well established the principle of phenomenology is to justify a smaller sample size rather than an explanation of how it was relevant to this study.
4. Table 1, Quality of dialogue row – Are there words missing in the Explanation column or could this be made clearer? It doesn’t feel like it quite makes sense. Is the intention to say that when there is stronger dialogue, a smaller sample size is required?
5. Table 1, Quality of dialogue row – The second mention of KG as the interviewer appears to be redundant and could be deleted.
6. Page 9, line 158 – This mention of the interview dates also appears to be redundant, given that this is also mentioned on page 7, lines 143-145.
7. Page 9, line 175 – Is the mention of KG as the interviewer again redundant in light of Table 1, or vice versa?
8. Page 9, line 176 – I would recommend adding a percentage to the mention of two participants to provide context in terms of the overall study size.
9. In the Results, the participants often make reference to past experiences and employment, yet they are being presented as representing their current position. I think it needs to be made clear that the scope of the interviews was not limited to their current position and the participants were free (and encouraged?) to reference past experiences outside of their current position. That diversity of experience is a good thing, but I think this context needs to be given in that the characteristics presented in the results are not 100% reflective of what is being discussed here. Likewise, comments are made about what other people are doing (e.g. page 26, line 353), so this should also be flagged as being within scope

Results

1. Please provide the total number of study participants upfront as part of the participant characteristics.
2. I recommend providing the exact percentages when mentioning patient characteristics because “most” is an ambiguous term that does not allow the reader to gauge exactly what proportion of the participants fit in any one category.
3. Page 12, lines 215­­‑216 – The numbers here add up to 24 compared with the N of 20. For clarity, I would suggest presenting these numbers as journal only, publishing group only and both rather than including the both group within each of the other subsets (i.e., counting 2 participants three times).
4. Page 12, lines 215­­‑216 – Subsets should be presented as lower case n, in line with convention (N being for the overall population) and I would also recommend presenting percentages alongside n numbers for additional context.
5. Page 13, lines 217–218 – n=11 is described as both half and almost half in the same sentence. Regardless, with such a small sample size, I would generally try and avoid approximations such as “half” because of the small sample size and how the sample was sourced, which means the generalisability of these statements is limited.
6. Table 3 – N=20 is in the table caption and table. It is only necessary to present this once, perhaps as ‘Characteristics (N=20)’ and then leaving the second column as n (%) (see next comment)?
7. Table 3 – Rather than two columns, I suggest considering presenting the data as n (%) in one column. This is easier to read and understand compared with two columns with numbers, which initially looks like two arms of a single study.
8. Table 3, journal publishers – I think this requires greater transparency because the Methods indicate that 5 publishers were targeted, yet an ‘Other’ category is introduced. This may need to be flagged as a possibility in the Methods.
9. Table 3, journal publishers – The ‘other’ publishers need to be identified. In the age of predatory journal publishers, it is extremely important to name these publishers, possibly in a footnote with n numbers for transparency about who was being interviewed.
10. Table 3, job title – If a n of 1 is provided for Senior Editor and Production Editor, then these should either be bundled in ‘Other’ and all six positions defined in a footnote, or the other positions spelt out, especially if any of them have an n ≥2.
11. Table 3, number of editorial decisions/week – Is the 50+ row necessary if it has a zero value and all the other values add up to 20?
12. Table 3, general – It would be good to have more granular information about the journals involved, for example, journal scope (e.g., general [*N Engl J Med*], speciality [*Blood*], sub-specialty [*Pediatr Blood Cancer*]; see page 20, line 362 as an example of where ‘specialty’ journals are mentioned and page 46, lines 810–811 where differing journal scope is discussed, without context being provided in the current characteristics table), publication frequency (weekly/monthly etc.), online only vs print, open access only vs pay for access, impact factor (probably categories, e.g., 0–2, 2–4, etc.) to provide context for who the participants are and what sort of journals they are representing because I think that is key to understanding the comments given. Each of these characteristics could have a major impact on decision making around PLSs.
13. Table 3, employment – Is it possible to outline how many are in paid employment versus voluntary roles. Are the two participants who represent both the publisher and journal publisher employees who at as journal editors, which seems to be becoming more common. That might need to be clarified for context. I apologise if this is somewhat redundant in the context comment 3, but it might help to distinguish academics from paid employees.
14. Quotes – Would it be possible to provide some context for each participant at first mention, such as ‘Participant 3 (Editor-in-Chief, general medicine journal)’. I think such detail is important framing for each quote. As noted in the Methods, biases will be present, so I think it is important for the reader to be made aware of some of these potential biases and the context within which these quotes are being made.
15. Page 19, lines 336 – I just want to check that “So, having a like, there’s no for them, maybe that they don’t…” is a correct transcription because it is very broken text and doesn’t seem to have a real flow of thought attached. Perhaps this could be deleted and just presented as “….communicating with….they don’t see a purpose….”
16. Page 28, line 527 – Please human in the loop is hyphenated here, but not on line 547. You may wish to make this consistent.
17. Page 33, lines 659–661 – Please state specific numbers rather than ‘a few’ and confirm whether these people with formal participants of this study or people outside of this study.
18. Page 37, lines 743–746 – I think this is intended to be italics,
19. Page 38, line 768 – When referring to ‘consumer support groups’ is this too broad? Should this refer to patient groups or healthcare consumer groups?

Discussion

1. Page 40, lines 824–825 – With reference to comment 12 on the Results, if commentary is to be provided on journals that did and did not publish PLSs, then this data should probably be included as a characteristic in Table 3.
2. Page 41, lines 834–836 – This reads as if PLSs are exclusively generated by the journals themselves, which is not correct. Some journals do develop PLSs internally, while others will offer a paid service to the Authors, but often it is up to the Authors themselves to generate the PLS. I think this should be clarified. I think there is also a greater discussion to be had around the challenges with PLSs as a journal editor, in particular around peer review, and ensuring that this occurs. It is a little bit of a rabbit hole, because you could also ask about who should be reviewing a PLS, but I think this goes to the point that the burden on Editors around PLSs is not about generating them, but how to handle them.
3. Page 41, lines 846–847 – Journals have plenty of information about the impact of PLSs, independently of tracking login details, although they don’t make it public about accesses to manuscripts and so on, so I question the statement about difficulty in assessing the impact of a PLS. Likewise, there is ample literature on the impact of PLSs on data dissemination, reader engagement and citations that could be cited when discussing the impact of PLSs (see Bennett & Slattery, 2023; Hoffberg et al., 2020; Ibrahim et al., 2017; Stahl-Timmins et al., 2019; West et al., 2020 as examples). This should also be considered when reviewing page 41, lines 846–849, which states that it is both difficult to asses impact and a more evidence-based approach to research should be performed because it arguably can and has been done already using some quite sophisticated study methodology.
4. General comment – It would be great to see if some positive suggestions could be provided about how some of the potential barriers may be overcome. With reference to point 3, there are plenty of benefits in publishing PLSs, so I wonder if any practical suggestions or ideas were floated during the interviews? If not, then maybe the fact that Editors are grappling with how to overcome some of these barriers is a key point to make in that a lack of intent is not the problem.

Conclusion

1. Page 41, line 852 – Should this be limited to ‘in peer-reviewed scientific articles’? PLSs can be generated via many means and by various parties, but this manuscript has a limited focus.
2. Page 41, lines 852–854 – As per Discussion comment 2, I would say that journals only generate a fraction of PLSs. Most are developed by the authors themselves, so I would suggest that this should refer to publishing, not producing, PLSs.
3. General comment – Are their barriers to PLSs being published, or a lack of incentives? We know there are benefits based on the literature, so is it actually a case of the incentives not being great enough?

References

1. Overall comment – The references should be styled consistently throughout the references section.
2. Reference 2 is missing an article number.
3. Reference 5 is missing an article number.
4. Reference 7 is missing an article number.
5. Reference 16 is a duplicate of reference 3.
6. Please provide the actual page number for reference 17, not the range of pages across the entire supplement.

Declarations of conflicting interest

1. This section must be rewritten to make appropriate disclosures upfront. The current format of offering no potential conflicts of interests, then listing exceptions is not acceptable. This should be rewritten detailing the relevant disclosures only. If any Author(s) have no disclosures, then this should be stated independently.
2. It is essential that the Authors who are journal editors indicate which journals they are an editor of because the reader must be given the opportunity to know which journal and which publishers may have a relationship with the Authors. If these are volunteer roles, this should also be clearly disclosed as such given that the Authors has disclosed no financial relationship with organisations with an interest in the work.
3. It would be helpful if the Authors could explicitly state that the journals they edit, and any related parties such as staff members of those journals, were not included in this study, presuming that was the case. If not, this should be disclosed.
