# Supplementary material for: What do editors of medical journals think about opportunities and barriers to advancement in the publication of plain language summaries? A qualitative analysis
Source: PLoS One. 2026 Mar 11;21(3):e0343058. doi: 10.1371/journal.pone.0343058 (PMC12978476; doi:10.1371/journal.pone.0343058)
Supplement: Attachment — Submitted filename: Response to Reviewers.docx [file pone.0343058.s006.docx]

**Author comments to reviewers – PONE-D-25-30641**

**REVIEWER ONE**

I believe that this manuscript is worthy of publication, but may some amendment, particularly around providing adequate data on the characteristics of the participants to support interpretation of the data, and also a more thorough review of the literature surrounding the impact of PLSs than is currently provided.

This qualitative analysis provides insight into the thoughts and impressions of peer-reviewed journal editors on the adoption of plain-language summaries (PLSs) by medical journals, which is a topic of ongoing debate in terms of how to encourage greater adoption of this innovative format by both journals and authors.

Overall, I think that the manuscript is deserving of publication and offers an opportunity to further contribute to the discussion about the implementation of PLSs. However, there are a few comments that I hope that Authors this manuscript may be able to consider and apply before I believe this manuscript would be fit for publication. For ease, I have simply commented by section rather than splitting my comments in major/minor categories below:

**Introduction**

Reviewer comment

Would it be possible to mention the heterogeneity of what may be considered a PLS? The term itself has become anything but plain language, given that the likes of the Good Publication Practice Guidelines 2022 offer a very broad definition of just about any derivative of a manuscript, while the lay person would probably consider a PLS to be a version of the abstract written in plain language. I think this is particularly relevant in that the impression I get is that this manuscript intends to focus on written PLSs, excluding video podcasts etc.

Author response

Thank you for your suggestion, which is pertinent given some of the findings from the study regarding non-text based formats. I have added some text to point out the various formats that are broadly considered ‘PLSs’ on Page 3.

*“Most commonly, PLSs are text-based; however, many journals offer alternative formats incorporating audio and visual elements, e.g., infographics”*

Reviewer comment

Page 10, line 16 – Is the Stoll reference intended to be replaced with a reference number?

Author response

Yes it should have been a reference number. I have corrected this oversight.

**Methods**

Reviewer comment

Were there any additional limitations on eligibility for this study? In particular, I think it might be pertinent to mention if the subjects could only represent journals publishing in English, given the multinational representation, and this seems to be mentioned in the Discussion on page 40, lines 828–829.

Author response

I agree that this is an important inclusion criteria. We did include this in the Methods section as one of our criteria on Page 6.

*“Works or volunteers for a journal that publishes research articles in English”.*

Reviewer comment

Table 1, Sample specificity row – Is sample density correct here, when referring to sample specificity? As a casual reader, I would interpret high sample density as suggesting a high proportion of journals who were eligible for this study were sampled.

Author response

I understand your concern. According to Malterud et al (2015), a sample specificity can be considered “dense” when the participants hold information, experience and properties relevant to the research question(s). As such, our sample could be characterized as sufficiently dense. We have added the following in the table for clarification:

*“Our sample specificity could be considered dense given that participants belonged to a specified target group while also exhibiting some variation within the experiences to be explored.”*

Malterud K, Siersma VD and Guassora AD (2015) Sample Size in Qualitative Interview Studies: Guided by Information Power. *Qualitative Health Research*. 26(13): 1753-1760. Available from: <https://doi.org/10.1177/1049732315617444>

Reviewer comment

Table 1, Established theory row – I’m not sure I 100% understand how the application in the current study links to the need for a smaller sample size. I guess I was kind of looking for an explanation of how well established the principle of phenomenology is to justify a smaller sample size rather than an explanation of how it was relevant to this study.

Author response

I have reworded the description ‘Established theory’ in the second column to help with clarity. It now reads “When stronger and more established theories are used, smaller sample size is required.”

This means that, as the theories of phenomenology and reflexive thematic analysis are well established, a smaller sample size is indicated.

Reviewer comment

Table 1, Quality of dialogue row – Are there words missing in the Explanation column or could this be made clearer? It doesn’t feel like it quite makes sense. Is the intention to say that when there is stronger dialogue, a smaller sample size is required?

Author response

Thank you for picking up this oversight. I have added the additional text, which will hopefully provide the necessary clarity.

*“When there is stronger dialogue in interviews, a smaller sample size is required.”*

Reviewer comment

Table 1, Quality of dialogue row – The second mention of KG as the interviewer appears to be redundant and could be deleted.

Author response

I agree and have made this deletion in Table 1.

Reviewer comment

Page 9, line 158 – This mention of the interview dates also appears to be redundant, given that this is also mentioned on page 7, lines 143-145.

Author response

I have removed the mention of interview dates on page 7.

Reviewer comment

Page 9, line 175 – Is the mention of KG as the interviewer again redundant in light of Table 1, or vice versa?

Author response

I appreciate that there might be some redundancy in this, however, I have kept the mention of KG as interviewer on Page 9 for two reasons. First, some readers may not have read Table 1 thoroughly. Second, it is accepted convention to note the initials of the research team when they are mentioned in the text, for transparency.

Reviewer comment

Page 9, line 176 – I would recommend adding a percentage to the mention of two participants to provide context in terms of the overall study size.

Author response

Thank you for this comment. I have added text at the beginning of this paragraph as well as the percentage of participants (2/20;10%) as suggested, to add clarity to our sample size.

*“We conducted 20 semi-structured interviews, guided by the first author (KG)”.*

*“For the two participants (2/20; 10%) who chose this option, we emailed the raw transcript within two days of their interview.”*

Reviewer comment

In the Results, the participants often make reference to past experiences and employment, yet they are being presented as representing their current position. I think it needs to be made clear that the scope of the interviews was not limited to their current position and the participants were free (and encouraged?) to reference past experiences outside of their current position. That diversity of experience is a good thing, but I think this context needs to be given in that the characteristics presented in the results are not 100% reflective of what is being discussed here. Likewise, comments are made about what other people are doing (e.g. page 26, line 353), so this should also be flagged as being within scope.

Author response

I agree with your point and appreciate that this was not included in the Methods section. I have added text to provide clarity to our approach with participants regarding their previous as well as current editorial experience.

*“During interviews, participants were encouraged to draw on any previous editorial experience to supplement that from their current position.”*

**Results**

Reviewer comment

Please provide the total number of study participants upfront as part of the participant characteristics.

Author response

I have added this to the Methods section on Page 10, line 172.

Reviewer comment

I recommend providing the exact percentages when mentioning patient characteristics because “most” is an ambiguous term that does not allow the reader to gauge exactly what proportion of the participants fit in any one category.

Author response

I have added frequency and percentages data.

*“Most participants were male (11/20; 55%), resided in either the United Kingdom or Australia (13/20; 65%), had worked as an editor for at least five years (15/20; 75%) and reported making up to 10 editorial decisions each week (10/20; 50%). Four (20%) participants were unsure how many editorial decisions they made per week.”*

Reviewer comment

Page 12, lines 215­­‑216 – The numbers here add up to 24 compared with the N of 20. For clarity, I would suggest presenting these numbers as journal only, publishing group only and both rather than including the both group within each of the other subsets (i.e., counting 2 participants three times).

Author response

I understand how that could be confusing, so I have taken your suggestion. The sentence now reads as “Most (N=17) participants represented a journal and 5 represented a publisher/publishing groups.”

Reviewer comments

Page 12, lines 215­­‑216 – Subsets should be presented as lower case n, in line with convention (N being for the overall population) and I would also recommend presenting percentages alongside n numbers for additional context.

Page 13, lines 217–218 – n=11 is described as both half and almost half in the same sentence. Regardless, with such a small sample size, I would generally try and avoid approximations such as “half” because of the small sample size and how the sample was sourced, which means the generalisability of these statements is limited.

Table 3 – N=20 is in the table caption and table. It is only necessary to present this once, perhaps as ‘Characteristics (N=20)’ and then leaving the second column as n (%) (see next comment)?

Table 3 – Rather than two columns, I suggest considering presenting the data as n (%) in one column. This is easier to read and understand compared with two columns with numbers, which initially looks like two arms of a single study.

Author responses

Thank you for your recommendations. I have amended Table 3 accordingly.

Reviewer comments

Table 3, journal publishers – I think this requires greater transparency because the Methods indicate that 5 publishers were targeted, yet an ‘Other’ category is introduced. This may need to be flagged as a possibility in the Methods.

Table 3, journal publishers – The ‘other’ publishers need to be identified. In the age of predatory journal publishers, it is extremely important to name these publishers, possibly in a footnote with n numbers for transparency about who was being interviewed.

Author response

I appreciate the need for transparency. As noted in the Methods, we were open to including journals from publishers other than those that we targeted specifically.

*“To engage with editors from journals that publish in the area of health literacy and health communication (given the relevance to PLSs), we included editors from these journals even if they didn’t publish PLSs.”*

Regarding the specific journals included in the “Other” category in Table 3, unfortunately authors are unable to provide any journals publishers specifically in this category as that would contravene the terms outlined in the participant consent form, which ensured participant anonymity would be protected. Journals in the “Other” category vary in size, however, all participants and the journals or publishers they represented were investigated prior to interviews to ensure they were not predatory in nature.

Reviewer comment

Table 3, job title – If a n of 1 is provided for Senior Editor and Production Editor, then these should either be bundled in ‘Other’ and all six positions defined in a footnote, or the other positions spelt out, especially if any of them have an n ≥2.

Author response

I appreciate the confusion over the way that the results for job titles have been presented. Unfortunately, more than one job title included in the sub-category of ‘Other’ is so specific that I could easily lead to identification of the participants. I have added a footnote to make this clear. There are no job titles in ‘Other’ with a frequency of >1.

*“These job titles have not been included because their specificity could lead to identification of the participants.”*

Reviewer comment

Table 3, number of editorial decisions/week – Is the 50+ row necessary if it has a zero value and all the other values add up to 20?

Author response

Although not necessary, the fact that it’s value is zero conveys useful information i.e., there appears to be a cap on the number of perceived editorial decisions per week. For that reason, I have left this row in Table 3.

Reviewer comment

Table 3, general – It would be good to have more granular information about the journals involved, for example, journal scope (e.g., general [*N Engl J Med*], speciality [*Blood*], sub-specialty [*Pediatr Blood Cancer*]; see page 20, line 362 as an example of where ‘specialty’ journals are mentioned and page 46, lines 810–811 where differing journal scope is discussed, without context being provided in the current characteristics table), publication frequency (weekly/monthly etc.), online only vs print, open access only vs pay for access, impact factor (probably categories, e.g., 0–2, 2–4, etc.) to provide context for who the participants are and what sort of journals they are representing because I think that is key to understanding the comments given. Each of these characteristics could have a major impact on decision making around PLSs.

Author response

I appreciate your request for the inclusion of this level of detail. Authors have not included detailed journal information to ensure that the journal in not identifiable by the reader, preserving the anonymity of the participants, as previously discussed.

Reviewer comment

Table 3, employment – Is it possible to outline how many are in paid employment versus voluntary roles. Are the two participants who represent both the publisher and journal publisher employees who at as journal editors, which seems to be becoming more common. That might need to be clarified for context. I apologise if this is somewhat redundant in the context comment 3, but it might help to distinguish academics from paid employees.

Author response

Unfortunately, this data was not collected, so we cannot report it.

Reviewer comment

Quotes – Would it be possible to provide some context for each participant at first mention, such as ‘Participant 3 (Editor-in-Chief, general medical journal)’. I think such detail is important framing for each quote. As noted in the Methods, biases will be present, so I think it is important for the reader to be made aware of some of these potential biases and the context within which these quotes are being made.

Author response

I appreciate that this distinction adds context to the participant examples/quotes. I cannot provide detailed participant information that could be considered identifiable, so I have included information that will add context to the participant examples/quotes without violating participant confidentiality. I have added the following information after each participant example/quote: editor/publisher and journal category. For example “…” Participant X (editor/general medical journal).

To keep this simple, journals have been categorised according to their primary scope. The journal categories are:

- General medical (n=6)
- Specialist medical (n=5)
- Allied health (n=3)
- Public health, health promotion, preventative medicine, healthcare policy (n=4)
- Health & medical education (includes health literacy & health communication journals) (n=5)

I have now added these journal categories to Table 4. Journal characteristics.

Reviewer comment

Page 19, lines 336 – I just want to check that “So, having a like, there’s no for them, maybe that they don’t…” is a correct transcription because it is very broken text and doesn’t seem to have a real flow of thought attached. Perhaps this could be deleted and just presented as “….communicating with….they don’t see a purpose….”

Author response

The quote is correct and transcribed verbatim. I have trimmed some of the unnecessary text as suggested to enhance sentence flow.

“*and that's the audience that you're communicating with….they don't see a purpose of having a plain language summary”.*

Reviewer comment

Page 28, line 527 – Please human in the loop is hyphenated here, but not on line 547. You may wish to make this consistent.

Author response

Thank you for being so thorough. I have removed the hyphens from the first mention of this phrase for consistency.

Reviewer comment

Page 33, lines 659–661 – Please state specific numbers rather than ‘a few’ and confirm whether these people with formal participants of this study or people outside of this study.

Author response

I have reworded this sentence to improve the clarity.

*“Several participants had with experience working with plain language summaries of publications (PLSPs), across more than one publishing group.”*

Reviewer comment

Page 37, lines 743–746 – I think this is intended to be italics,

Author response

Thank you – this has been fixed.

Reviewer comment

Page 38, line 768 – When referring to ‘consumer support groups’ is this too broad? Should this refer to patient groups or healthcare consumer groups?

Author response

Authors have used the term consumer here for consistency. As per my previous response about the use of the term consumer, this term is appropriately descriptive.

**Discussion**

Reviewer comment

Page 40, lines 824–825 – With reference to comment 12 on the Results, if commentary is to be provided on journals that did and did not publish PLSs, then this data should probably be included as a characteristic in Table 3.

Author response

This is a good point. I created a new Table (Table 4) and have added this information, as well as that for journals for which PLSs were and were not mandatory. I included the latter as this was also noted in the Discussion.

Reviewer comment

Page 41, lines 834–836 – This reads as if PLSs are exclusively generated by the journals themselves, which is not correct. Some journals do develop PLSs internally, while others will offer a paid service to the Authors, but often it is up to the Authors themselves to generate the PLS. I think this should be clarified. I think there is also a greater discussion to be had around the challenges with PLSs as a journal editor, in particular around peer review, and ensuring that this occurs. It is a little bit of a rabbit hole, because you could also ask about who should be reviewing a PLS, but I think this goes to the point that the burden on Editors around PLSs is not about generating them, but how to handle them.

Author response

I appreciate your concerns about this highly nuanced area. Regarding the peer review of PLSs, authors noted the challenges discussed by participants in Theme 1.4 Going over and above in the context of resource limitations.

*“Given the multiple tasks editors had to juggle, once put in place, processes involving PLSs such as author instructions and peer review, were rarely reviewed.*

*Participant 3: Are the headings that we have there the right ones? I don't know. But you know, we've got a whole, I've got a whole process of things that I'm doing at the moment, and this serves a function. So, we've decided to leave them as they are at the moment.”*

This was also discussed more fully in Theme 2.3 Peer reviewers.

I note that some journals do offer a paid service to authors for PLSs, however, participants noted that this was rarely used due to cost. The topic of who should write a PLS and the associated costs is discussed in Theme 2 – Whose job is it anyway?

Reviewer comment

Page 41, lines 846–847 – Journals have plenty of information about the impact of PLSs, independently of tracking login details, although they don’t make it public about accesses to manuscripts and so on, so I question the statement about difficulty in assessing the impact of a PLS. Likewise, there is ample literature on the impact of PLSs on data dissemination, reader engagement and citations that could be cited when discussing the impact of PLSs (see Bennett & Slattery, 2023; Hoffberg et al., 2020; Ibrahim et al., 2017; Stahl-Timmins et al., 2019; West et al., 2020 as examples). This should also be considered when reviewing page 41, lines 846–849, which states that it is both difficult to assess impact and a more evidence-based approach to research should be performed because it arguably can and has been done already using some quite sophisticated study methodology.

Author response

I acknowledge that PLSs in forms other than text-based are more frequently published on social media, with the impact recorded in several studies, such as those you cited. I have added text to the Discussion to acknowledge this point.

Regarding the tracking of text-based PLSs, unless they are published via social media, they are difficult to track. Most PLSs are embedded within journal articles rather than stored in searchable, centralised repositories e.g., Cochrane Library.

In terms of accessibility, and searchability, unlike plain language summaries of publications (PLSP), PLSs do not have a unique DOI. To make PLSs more discoverable, in early 2019, PubMed introduced a new function whereby journal publishers can tag PLSs with the XML tag 'plain-language-summary' in the Other Abstract field (Collins, 2019). This will allow the PLSs to be indexed on PubMed, where they are freely available to millions of users daily (Rosenberg et al., 2023b). Rosenberg and colleagues (2023) measured the prevalence of the XML tag in PubMed records to determine the uptake by journal publishers. After analysing 31,817,472 PubMed records, they found that only 3217 (0.01%) had an XML tag 'plain language summary' in the Other Abstract field, with approximately half of these (1644, 51%) being published in 2021 (Rosenberg et al., 2023). Of these 3217 records, 470 (14.6%) were incorrectly tagged (Rosenberg et al., 2023). Although introduced over five years ago, the low adoption rate of this metatag may be due to a lack of awareness or understanding by journal publishers about how to use it correctly.

The above paragraph is not additional text added to the manuscript. I have added this by way of a more thorough explanation, and provided references to support my statements.

Collins, M. PubMed Updates February 2019. (2019). *NLM Tech Bul*. Jan-Feb;(426)e4. Available from: <https://www.nlm.nih.gov/pubs/techbull/jf19/jf19_february_pubmed_updates.html>

Rosenberg, A., Baróniková, S., Gattrell W., Ghildyal, N., Koder, T., Koskenkorva, T., Liew, A., Narayan, R., Osorio, J., Philippon, V., Shane, M., Skobe, C., & Wager, K. (2023). Assessing PubMed metatag usage for plain language summary discoverability. [Poster presentation]. 2023 European meeting of the International Society for Medical Publication Professionals, London, United Kingdom. Available from: <https://peerreviewcongress.org/abstract/assessing-pubmed-metatag-usage-for-plain-language-summary-discoverability/>

Reviewer comment

General comment – It would be great to see if some positive suggestions could be provided about how some of the potential barriers may be overcome. With reference to point 3, there are plenty of benefits in publishing PLSs, so I wonder if any practical suggestions or ideas were floated during the interviews? If not, then maybe the fact that Editors are grappling with how to overcome some of these barriers is a key point to make in that a lack of intent is not the problem.

Author response

I am unclear which point you are referring to, however participants made numerous suggestions for improvement in the publication of PLSs. As noted in the Results, editors do not always have the autonomy to implement the changes they want, or in the timeframe they believe appropriate. That is the key focus of Theme 1 When good intentions clash with practical realities.

**Conclusion**

Reviewer comment

Page 41, line 852 – Should this be limited to ‘in peer-reviewed scientific articles’? PLSs can be generated via many means and by various parties, but this manuscript has a limited focus.

Author response

I believe you are referring to this statement – the first sentence in the conclusion. “PLSs have the potential to meaningfully engage people with trustworthy research.”

I understand your point, however, I believe the context for this study is clear i.e., of PLSs from peer reviewed journal articles. Also, this statement, although broad, applies to PLSs in general, regardless of their source.

Reviewer comment

Page 41, lines 852–854 – As per Discussion comment 2, I would say that journals only generate a fraction of PLSs. Most are developed by the authors themselves, so I would suggest that this should refer to publishing, not producing, PLSs.

Author response

I understand your point, however this statement is reflective of participant comments during interviews, during which they all expressed enthusiasm for the production and publication for PLSs, independent of who writes them. I have added the words “and publication” to improve the clarity of this sentence.

*“While many individuals in health and medical publishing are focused with enthusiasm on the production and publication of PLSs, barriers often surmount the desire to nudge innovation and adapt to global trends.”*

Reviewer comment

General comment – Are their barriers to PLSs being published, or a lack of incentives? We know there are benefits based on the literature, so is it actually a case of the incentives not being great enough?

**References**

Reviewer comments

Overall comment – The references should be styled consistently throughout the references section.

- Reference 2 is missing an article number.
- Reference 5 is missing an article number.
- Reference 7 is missing an article number.
- Reference 16 is a duplicate of reference 3.
- Please provide the actual page number for reference 17, not the range of pages across the entire supplement.

Author responses

- The article number for reference 2 is included. It is 22.
- The article number for reference 5 is included. It is 121.
- I have removed reference 16 from the reference list.
- I have corrected this. I noticed that the article link was incorrect, so I have corrected this also.

**Declarations of conflicting interest**

Reviewer comment

This section must be rewritten to make appropriate disclosures upfront. The current format of offering no potential conflicts of interests, then listing exceptions is not acceptable. This should be rewritten detailing the relevant disclosures only. If any Author(s) have no disclosures, then this should be stated independently.

Author response

I apologise for the irregularity in my response to this section. I have added corrected Declarations of conflicting interest section with the following addition:

*“Danielle Muscat is Editor in Chief of the journal Health Literacy and Communication Open and Kirsten McCaffery is Editor in Chief of the journal Patient Education and Counselling.”*

Reviewer comment

It is essential that the Authors who are journal editors indicate which journals they are an editor of because the reader must be given the opportunity to know which journal and which publishers may have a relationship with the Authors. If these are volunteer roles, this should also be clearly disclosed as such given that the Authors has disclosed no financial relationship with organisations with an interest in the work.

It would be helpful if the Authors could explicitly state that the journals they edit, and any related parties such as staff members of those journals, were not included in this study, presuming that was the case. If not, this should be disclosed.

Author response

As noted above, I have updated the Declarations of conflicting interest section to include the journals for which authors hold editorial positions.

**REVIEWER TWO**

Congratulations to the authors for selecting to investigate such a relevant topic for the current times. It is a complex topic and involves a relatively unreachable participant group, this deserves acknowledgement. I believe the insights from this paper will be a fantastic starting point to dig deeper into systemic challenges that plague the publishing industry, when it comes to offering PLSs.

Here are my recommendations.

**Introduction**
Reviewer comment

Well-written in the context of the current scope of the study. The only suggestion I would have is to assess whether this scoping review can complete the landscape sweep the authors aim to achieve. doi: 10.1007/s40271-024-00700-y

Author response

Thank you for your suggestion. I am aware of this paper titled “Practices and Barriers in Developing and Disseminating Plain‑Language Resources Reporting Medical Research Information- A Scoping Review”. Authors did not include it as a reference in this manuscript because it refers to plain language resources (PLRs) generally, not PLSs specifically. Although the paper notes that the most frequent terms used to denote PLRs were lay summary and plain language summary, the study located 26 terms for PLRs, some of which appear to be dissimilar to text-based PLSs from health and medical journals (the context for this study), to warrant the inclusion of this reference e.g., clinical trial results summary, clinical trial results, significance statement, translational abstracts, trial results summary (Fig 3). <https://doi.org/10.1007/s40271-024-00700-y>

**Methods**
I do have a few major questions on this section.

Defining criteria for applying the concept of information power: study aim, sample specificity, established theory, quality of dialogue, analysis strategy

Reviewer comment

Do the authors feel that the sample specificity was sufficiently narrow to support using information power rather than saturation?

Author response

I understand your concern. According to Malterud et al (2015), a sample has dense specificity when the participants hold information, experience and properties relevant to the research question(s). As such, our sample could be characterized as sufficiently dense. Also, the use of information power rather than data saturation to determine sample size is consistent with Braun and Clarke’s reflexive thematic analysis, an approach authors adopted throughout this study.

Malterud K, Siersma VD and Guassora AD (2015) Sample Size in Qualitative Interview Studies: Guided by Information Power. *Qualitative Health Research*. 26(13): 1753-1760. Available from: <https://doi.org/10.1177/1049732315617444>

Braun, V & Clarke, V. (2021) “To saturate or not to saturate? Questioning data saturation as a useful concept for thematic analysis and sample-size rationales”. *Qualitative research in sport, exercise and health,* 13(12), 201-216. Available from: <https://doi.org/10.1080/2159676X.2019.1704846>

Reviewer comment

Phenomenological analysis or reflexive thematic analysis involves exploration of lived experiences, which is rightly adopted by the authors. However, that doesn't offer the established theory for analysis, that the authors mention. It would be important to understand the rationale for this unique approach.

Author response

Reflexive thematic analysis is a methodological approach and a data analysis method. Please refer to “Table 2. Steps in reflexive thematic analysis”, in which authors outline the steps taken during data collection and analysis.

Reviewer comment
Was any software such as MaxQDA used?

Author response

We did not use any software to analyse data.

Reviewer comment
Was a coding tree developed and used for cross transcript analyses?

Author response

As discussed in the Methods section, “Our approach involved the steps included in Table 2. Although we followed these steps in the order below, reflexive thematic analysis is not intended to be a linear process [23]. We moved between assigning codes to theme development several times as we interpreted the data at increasingly deeper levels. KG and DM were involved in data analysis.

Authors did not develop a coding tree for cross transcript analysis, as this practice is not aligned with reflexive thematic analysis.

Reviewer comment
How were disagreements resolved?

Author response

It would not be relevant to report how disagreements are resolved because the practice of resolving disagreements is consistent with a positivist approach, which is not aligned with reflexive thematic analysis, in which researchers are not seeking to discover one ultimate truth.

Reviewer comment

Also, I'd recommend using the COREQ (COnsolidated criteria for REporting Qualitative research) checklist to make the reporting more robust.

Author response
I appreciate the need to provide a reporting checklist. I did provide a completed Standards for Reporting Qualitative Research (SRQR) checklist as an attachment to this paper. Perhaps this was not provided to peer reviewers.

Reviewer comment
Perhaps I missed this but what was KMcC's role in this whole interview and coding process? I'm being mindful of the contributions that qualify for authorship per ICMJE criteria.

Author response

I appreciate your concerns regarding authorship qualification according to ICMJE criteria. I have amended the authorship contributions in the submission portal to reflect the contribution of each author.

**Results**
Very well written and embeds the quotes from the participants effectively to support the insights drawn.

Author response

Thank you for your comment. We were fortunate to have such rich interview data from which to draw to help illustrate the study themes.

Reviewer comment
11 to 9 is perhaps not majority for males?

Author response

I appreciate the need for precision when reporting quantitative data. Although it not a statistically significant difference, 11/20 represents 55%. According to the Merriam-Webster dictionary, this represents a majority, in which a majority is a number or percentage equalling more than half of a total.

<https://www.merriam-webster.com/dictionary/majority>

Reviewer comment
Was any data collected to understand the exposure of participants to specifically PLS-related decision-making?

Author response

I agree that this is an important topic for discussion, as it is fundamental one of the study aims. Questions that relate to decision making and PLSs were usually broached during the interviews after a short introduction.

In case the interview guide (S1_Text) wasn’t made available to reviewers, I have included below the questions that pertain the PLS-related decision making.

Taken from S1_Text – Interview Guide

***Decision making and how that could impact changes to PLSs***

- Can you tell me about your role as a journal editor?
- Can you tell me about an experience you have had with PLSs?
- When it comes PLSs, who makes decisions about them?
  - Length
  - Content
  - Structure
  - Format i.e., text-based only or graphical/video options
- Have you faced any barriers to the implementation of PLSs?

Reviewer comment
As some participants represented more than one entity, does this make it challenging to analyze their input, as to whether their approach and experiences varied across the journals?

Author response

I appreciate that the varied background of participants may seem at times confusing. We did not seek to compare experiences across journals, so analysing data from participants with experience from multiple sources did not prove problematic. Participants clearly identified the journal or organisation they were referencing when making comments during interviews.

Reviewer comment
For the thematic analysis reporting, it would be even more impactful if the participants are labelled by which group they're representing, editors or publishers and, whether their journal publishes PLSs.

Author response

I appreciate the value of this information. I have to all participant comments/quotes whether the participant represents an Editor or Publisher, and created Table 4 which provides journal detail as requested.

Reviewer comments
Curious as to whether these topics came up during to discussions:
any experiences shared about this perceived "pain" of developing PLSs vs other formats such as visual abstracts?
1. whether they worry about an imbalance b/w industry-funded vs academic research PLSs?
2. making available or the utility of already available PLS author guides?
3. about some publishers developing PLSs themselves?
4. how to ensure that PLSs maintain a fair and balanced narrative?
5. how to make PLSs more searchable and accessible?

Author responses

Some of these topics were mentioned by some participants, and we included many of these topics in our themes.

1. This topic was mentioned in the context of resource limitation, with a couple of participants noting that industry funded research often has the support of a large research team, which includes a specialist medical writer who can write a high quality PLS.
2. In S1_Text – Interview Guide, there was a series of questions the relate to author guidelines for writing PLSs, so this area was discussed.

Taken from S1_Text – Interview Guide

***PLS guidelines***

- Does your journal have any guidelines to support authors in writing PLSs?
- Who developed them? Can you tell me a bit about that process?
- Was there any end-user input in their development?
- When were they last reviewed?

As noted in Theme 1.4 ‘Going over and above in the context of resource limitation’, authors note “Given the multiple tasks editors had to juggle, once put in place, processes involving PLSs such as author instructions and peer review, were rarely reviewed.

*Participant 3: Are the headings that we have there the right ones? I don't know. But you know, we've got a whole, I've got a whole process of things that I'm doing at the moment, and this serves a function. So, we've decided to leave them as they are at the moment.”*

3. This topic was covered and discussed in Theme 2 Whose job is it anyway?
4. This topic was not mentioned by participants.
5. This topic was covered in detail and discussed under Theme 5.2 Novel dissemination routes

Reviewer comment
Consumers: Technically an apt term, but in the context of PLSs as these could be patients, general public, etc, could there be a different term such as intended audience, end-user?

Author response

As there many apt terms, we decided to choose one of the most recognisable terms; consumer. A previous qualitative study by the authorship team of people who are high users of PLS, showed that patient is not a term favoured by this cohort. General public as term is too vague and not entirely accurate. There is contention in the field as to who the intended versus actual audience of PLSs is, a topic raised by this study. Therefore, intended audience would not be relevant, nor accurate. As there are many end-users of PLSs such as health professionals, non-specialist researchers, policy makers, consumer health organisations, funding bodies, the term end-user would not be sufficiently narrow to accurately reflect those involved in research co-design.

Gainey KM, McCaffery K and Muscat D. Perspectives of people with chronic illness about plain language summaries: a qualitative analysis. Health Promot Int 2025;40(2). Available from: <https://doi.org/10.1093/heapro/daaf044>

**Discussion**
Reviewer comment
Overall, this section can be improved. There is a wealth of insights in the Results section, which deserve more contextualization, to highlight the novelty of this research. For instance, the potential contradiction b/w editors of journals which offer PLSs vs those which don't. This is perhaps even more relevant in the context of perceived barriers.

Author response

I appreciate that this might appear to provide insight, however, the decision to publish PLSs was not always made by the editors. In most cases, it was a decision made by the Publisher and applied to all journals in their library.

This comment from Participant 15 encapsulates this attitude from publishers.

*“Participant 15: I don't think they [the publisher] see the journal, as even though it's open access as this public facing. I don't think they've moved, progressed to that level.”*

Authors discussed this finding in Theme 1.3 Uniformity and Autonomy

*“A few of the participants were associated with journals that did not produce PLSs and shared their thoughts on why this was the case. One person suggested that the actions of the journal demonstrated that they saw no purpose in including PLSs in their journal because their only audience was other professionals i.e. medical professionals and researchers not experts in the particular field covered by the journal. For another participant, the decision to not include PLSs was a lack of prioritisation. Both participants thought their respective journals were missing out on an opportunity to reach a broader audience by not including PLSs, especially when publishing open access.”*

Reviewer comment
I also wonder whether the study can be labelled as comprehensive, given the qualitative nature and typical lack of generalizability that comes with it. Detailed for sure, high value no doubt.

Author response

Generalisability of findings is not an approach that is consistent with reflexive thematic analysis, so that concern is not well founded. In terms of comprehensiveness, a review of S1_Text Interview Guide and the study themes might help to reassure you on this point. However, in an effort to address your concerns, I have reworded the sentence as follows.

*“This in-depth qualitative study provides novel insights into the decision-making process that govern the publication of PLSs, covering barriers, facilitators and attitudes of journal editors towards PLSs.”*

Reviewer comment
Restrictions around language (English only) and no specific consideration to include journals which publicize themselves as patient experience or participatory science journals - both are easy-to-identify limitations. The implications should be discussed under Discussions given that one of the main aims of PLSs is accessibility and inclusiveness.

Author response

I have added text to the Discussion to address this point.

*"While we purposively engaged editors from journals that publish in the area of health literacy and health communication (given the relevance to PLSs), we did not purposively sample for include journals which publicise themselves as patient experience or participatory science journals in the same way".*

Since we did not include the titles of any of the included journals, the statement that we did not include any journals “which publicize themselves as patient experience or participatory science journals” cannot be determined by the reader. Authors note in Methods the following:

*"To engage with editors from journals that publish in the area of health literacy and health communication (given the relevance to PLSs), we included editors from these journals even if they didn’t publish PLSs.“*
