# Supplementary material for: What do editors of medical journals think about opportunities and barriers to advancement in the publication of plain language summaries? A qualitative analysis
Source: PLoS One. 2026 Mar 11;21(3):e0343058. doi: 10.1371/journal.pone.0343058 (PMC12978476; doi:10.1371/journal.pone.0343058)
Supplement: Attachment — Submitted filename: Responses to reviewers.docx [file pone.0343058.s007.docx]

**Author responses to reviewers – revision 2**

**Reviewer #1:**

I appreciate the Authors making the effort to incorporate a large number of changes.

While I don't think that the additional information requested would be sufficient to allow the potential identification of the interviewees, I respect that the Authors are in the best position to make that judgement and how the manuscript is presented is their prerogative.

Therefore, I look forward to seeing this published in due course.

Author response: Thank you for your comment and your understanding the judgement of authors to not disclose the additional information that was requested on the basis of protecting the identification of study participants. Authors gave your request consideration, however, we were unable to justify the inclusion of the information requested. Authors apologise for overlooking one of your comments from the previous revision and will ensure it is addressed in this revision.

**Reviewer #3:**

Thank you for the detailed revisions and point-by-point responses. Overall, I think you have addressed the key concerns from the previous round. In particular, you clarified the heterogeneity of what can be considered a PLS and added text acknowledging non-text formats, which better aligns the scope and findings. You also strengthened methodological transparency by explicitly stating the English-language eligibility criterion, and improved interpretability of participant characteristics by adding frequencies/percentages and correcting presentation issues. The addition of a new table that captures whether PLSs are published or mandatory also responds well to prior suggestions. Lastly, the conflict of interest section appears to have been revised to reflect the requested clarity in disclosure.

Author response: Thank you for confirming that the changes made to the manuscript addressed your previous concerns. It is helpful to know that the inclusion of the additional information as requested enhanced the methodology and the presentation of findings in the manuscript.

**Reviewer #4:**

The comments were attached as a word document with tracked changes. Also, a critical appraisal checklist for qualitative studies was used as a tool during the review.

All pervious comments to the authors were addressed except one. It was added as a comment at the end of the manuscript file just before the references heading.

Author response: Thank you for providing detailed comments to this revised manuscript. As comments included in a tracked changes document, I will address each one, referencing page and line numbers from that same tracked changes document.

Reviewer (#4) comment 1

Page 1, Line 1 - Maybe change to ‘editorial staff’ since the recruited subjects include associate editors and editorial staff members, not only editors (lines 105, 109)

Author response

Thank you for this suggestion, however, authors are satisfied with the title.

Reviewer (#4) comment 2

Page 6, Line 122 - Upon checking the authors’ list for this reference, I could not point out a common author with the list of authors of this manuscript. Are you sure this is the correct reference/reference number?

If yes, please replace ‘our’ with ‘a’

Author response 2

Thank you for pointing out this oversight. We have amended this to correctly state reference 16, instead of 15.

Reviewer (#4) comment 3

Page 10, Line 174 - If the first author (KG) was involved in both the anonymisation and data analysis (line 190), what is the point of anonymisation?

I mean KG would be able to identify the source of the transcripts and thus preclude the benefits of blind analysis (analysis bias)

Author response 3

Authors do not state that the analysis was conducted as a blind analysis. Data was anonymised because it was uploaded to a public data repository.

Reviewer (#4) comment 4

Page 11, Line 188 - If the first author (KG) was involved in both the anonymisation (line 176) and data analysis, what is the point of anonymisation?

I mean KG would be able to identify the source of the transcripts and thus preclude the benefits of blind analysis (analysis bias).

Author response 4

Please see response for comment 3.

Reviewer (#4) comment 5

Page 12, Line 213 - Why have you not implemented follow-up queries to the participants to remove this category and divide the 4 into the previous categories? (This comment referred to the statement in the Results “Four (20%) participants were unsure how many editorial decisions they made per week.”)

Author response 5

The fact that four editors were unsure of how many editorial decisions they made per week was a valid finding. To probe this further with those four participants would have potentially introduced bias, unless the interviewer probed each response from each participant. During interviews, participants noted that the number of editorial decisions varied from week to week, some participants stating that they could not accurately recall how many they made with any degree of accuracy.

Reviewer (#4) comment 6

Page 15, Table 3 - This category can be removed and another footnote added to the previous category (20-50) to explain that 50 (or less) was the upper limit of the decisions per week in the study sample. (This comment relates to ‘Approximate number of editorial decisions/week’ and the finding of zero participants recording 50+/week.

Author response 6

As for the previous response, a finding of zero is a valid finding and worthy of reporting. With four participants stating that they made 20-50 decisions/week, to not include the responses for 50+/week would appear less than comprehensive. Including this value showed that the option of 50+ was included in the survey as a possible response, providing a clear ceiling of 50 as the upper value of editorial decisions/week, avoiding ambiguity.

Reviewer (#4) comment 7

Page 15, Table 4 - Why have you not implemented follow-up queries/investigations to remove this category and assign this journal into one of the previous categories? (This relates to the findings for ‘Journal publishes a PLS).

Author response 7

I appreciate the confusing nature of this finding, so have added a footnote to Table 4 to clarify. This participant could not recall this information and authors were unable to verify it because it related to an archived journal for which the author instructions were no longer available.

“Participant could not recall this information and since the journal was archived, the author instructions were not available to reference.”

Reviewer (#4) comment 8

Page 15, Table 4 - Why have you not implemented follow-up queries/investigations to remove this category and assign this journal into one of the previous categories? (This relates to the findings for ‘PLS is mandatory and reporting of “unsure’ for one participant).

Author response 8

Please see response to comment 7.

Reviewer (#4) comment 9

Page 16, Table 4 - I agree with reviewer no.1 comment regarding adding other rows to this table to show:

1. The number and percentage of journals in the study sample that offers an open access option (this would include both open access only and hybrid journals). The other category would be pay for access.

2.  The number and percentage of journals by publication frequency (weekly, monthly, ...)

I believe that these are important two characteristics of the journals’ sample that would be relevant to the discussion section and the conclusions of this study, particularly pertaining to the readership data headlines.

Author response 9

Please note my previous response to reviewer 1. The position of the authors remains unchanged.

I appreciate your request for the inclusion of this level of detail. Authors have not included detailed journal information to ensure that the journal in not identifiable by the reader, preserving the anonymity of the participants, as previously discussed.

Also, the inclusion of this information has limited bearing on the decision making of editors or publishers when it comes to PLSs

Reviewer (#4) comment 10

General comment:

I am repeating one of the interesting questions raised by reviewer no.1 that was not addressed by the authors:

“Are their barriers to PLSs being published, or a lack of incentives?

We know there are benefits based on the literature, so is it actually a case of the

incentives not being great enough?”

It would be helpful if the answer to this point was addressed in the discussion section

Author response 10

Thank you for bringing this oversight to our attention.

The issue of incentives was not discussed by participants and it was not a topic listed on the interview guide. Authors agree that there are benefits to the publication of PLSs, however, have not discussed the topic of incentives as the barriers to the more widespread adoption and publication of PLSs by scholarly publishers and journals are more nuanced than incentives, as can be seen by the thematic analysis in this manuscript.
